# High Glucose Aggravates Cholesterol Accumulation in Glomerular Endothelial Cells Through the LXRs/LncRNAOR13C9/ABCA1 Regulatory Network

**DOI:** 10.3389/fphys.2020.552483

**Published:** 2020-10-19

**Authors:** Yao Wang, Shumin Xiao, Saijun Zhou, Rui Zhang, Hongyan Liu, Yao Lin, Pei Yu

**Affiliations:** NHC Key Laboratory of Hormones and Development, Tianjin Key Laboratory of Metabolic Diseases, Chu Hsien-I Memorial Hospital & Tianjin Institute of Endocrinology, Tianjin Medical University, Tianjin, China

**Keywords:** diabetic nephropathy, GEnCs, ABCA1, LncRNAOR13C9, cholesterol accumulation

## Abstract

**Background:**

The underlying mechanisms by which diabetes and dyslipidemia contribute to diabetic nephropathy (DN) are not fully understood. In this study, we aimed to investigate the role of high glucose (HG) on intracellular cholesterol accumulation in glomerular endothelial cells (GEnCs) and its potential mechanism.

**Methods:**

Oil red O staining, RT-qPCR, Western blotting, and immunocytofluorescence analyses were used to determine cholesterol accumulation and the expressions of LXRs and ABCA1 in GEnCs under high cholesterol (HC) and/or HG conditions, and the effect of these treatments was compared to that of low glucose without adding cholesterol. LncRNA microarrays were used to identify a long non-coding RNA (LncRNA OR13C9), of which levels increased in cells treated with the LXR agonist, GW3965. Fluorescence *in situ* hybridization (FISH) was conducted to confirm subcellular localization of LncOR13C9 and a bioinformatics analysis was used to identify competing endogenous RNA (ceRNA) regulatory networks between LncOR13C9 and microRNA-23a-5p (miR-23a-5p). Gain and loss of function, rescue assay approaches, and dual-luciferase reporter assay were conducted to study interactions between LncOR13C9, miR-23a-5p, and ABCA1.

**Results:**

We showed that HG could decrease the response ability of GEnCs to cholesterol load, specifically that HG could downregulate LXRs expression in GEnCs under cholesterol load and that the decrease in LXRs expression suppressed ABCA1 expression and increased cholesterol accumulation. We focused on the targets of LXRs and identified a long non-coding RNA (LncOR13C9) that was downregulated in GEnCs grown in HG and HC conditions, compared with that grown in HC conditions. We speculated that LncRNAOR13C9 was important for LXRs to increase cholesterol efflux via ABCA1 under HC. Furthermore, using gain of function, loss of function, and rescue assay approaches, we showed that LncOR13C9 could regulate ABCA1 by inhibiting the action of miR-23a-5p in the LXR pathway. Furthermore, dual-luciferase reporter assay was conducted to study the interaction of LncOR13C9 with miR-23a-5p.

**Conclusion:**

Overall, our study identified the LXRs/LncOR13C9/miR23A-5p/ABCA1 regulatory network in GEnCs, which may be helpful to better understand the effect of HG on cholesterol accumulation in GEnCs under cholesterol load and to explore new therapeutic tools for the management of DN patients.

## Introduction

Diabetic nephropathy (DN) is one of the most common and serious microvascular complications of diabetes (Choby, 2017). Epidemiological surveys have found that it is expected that the number of global diabetes patients will reach 642 million by 2020 (Choby, 2017; Zhao et al., 2018). Due to the increase in the number of diabetic patients, DN has become the most common cause of end-stage nephropathy (Zhao et al., 2018). At present, although many theories have been put forward regarding the pathogenesis of DN, its specific mechanism is still unclear. As early as in the 1930s, some scholars discovered the existence of adipose deposition in kidney tissue of DN patients after autopsy (Murea et al., 2010; Shapiro et al., 2011). It indicated that the damage caused by adipose deposition on renal cells played a role in the progression of DN and chronic renal failure. Subsequently, lipotoxicity, a theory in which accumulation of excess lipid in non-adipose tissue leads to cell dysfunction and potential cell death was proposed (Kohlwein and Petschnigg, 2007). Many studies have found that lipotoxicity may be involved in the occurrence and development of non-alcoholic fatty liver disease, atherosclerotic cardiovascular disease, islet cell dysfunction, diabetic macrovascular, and microvascular complications (Gaemers et al., 2011; Plötz et al., 2016; Hennessy et al., 2019). At present, we know that the maintenance of lipid homeostasis is essential for human health and that abnormal lipid metabolism and accumulation, including that of cholesterol and triglycerides, result in disease states. The removal of excess cholesterol from cells and its delivery to the liver are important in protection from pathologic cholesterol accumulation in tissues (Siddiqi et al., 2015). Intracellular cholesterol accumulation in the kidney is a characteristic of high fat-induced kidney damage (Declèves et al., 2014), and an understanding of the regulatory mechanism of cholesterol at the cellular level is necessary.

To our knowledge, liver-X-receptors (LXRs) play a key role in the regulation of genes by controlling the response to excess cholesterol, particularly that of ATP binding box transporter A1 (ABCA1) (Lee and Tontonoz, 2015). ABCA1 is a membrane protein that is highly expressed in the liver, kidneys, other organs, and tissues (Oram and Lawn, 2001; Wellington et al., 2002). It functions as a primary gatekeeper for the elimination of excess free cholesterol from cells to lipid-free apoA-I, resulting in the formation of nascent high-density lipoprotein (HDL) (Attie et al., 2001; Oram and Lawn, 2001). The results of Zhu et al. showed that, compared with macrophages of wild type mice, macrophages of ABCA1 knock-out (KO) mice showed a >95% reduction in ABCA1 protein levels and a significant increase in free cholesterol (FC), and that excess FC accumulation could induce cytotoxicity and apoptosis of macrophages (Zhu et al., 2008). Moreover, Liu and colleagues found that ABCA1 participates in the outflow of cholesterol from renal cells, and its expression is downregulated in HG environments (Liu et al., 2018).

The annotation of sequencing results showed that less than 2% of the human genome is composed of protein-coding genes and that a majority were non-coding genes that produce a large number of non-coding transcripts, including cirRNAs, microRNAs, and LncRNAs (Talkish et al., 2014; Boivin et al., 2018; Lawrie et al., 2018). LncRNAs are more than 200 nucleotides long and they can exert their functions by forming lncRNA-protein, lncRNA-DNA, and lncRNA-RNA interactions. Although it is estimated that the human genome includes >10,000 lncRNAs, fewer than 5% of all lncRNAs have been functionally characterized (Palazzo and Lee, 2015; Schmitz et al., 2016). In addition, recent studies have found that LncRNAs exert regulatory functions involved in the occurrence and development of diabetes and control of cellular cholesterol metabolism (Li D. et al., 2017; Das et al., 2018). For example, several lncRNAs have been found to promote or inhibit the functions of regulatory genes involved in cholesterol synthesis and efflux, including LeXis, MeXis, and CHROME (Sallam et al., 2016, 2018; Hennessy et al., 2019).

DN is a diabetic microvascular complication, and dysfunction of glomerular endothelial cells (GEnCs) is involved in this pathological process (Matsuda et al., 2018). Increasing evidence has shown that GEnCs are an integral part of the glomerular filtration barrier, and that GEnCs injury or dysregulation of cross-talk between GEnCs and podocytes contributes to the development and progression of DN (Broekhuizen et al., 2010; Byron et al., 2014; Herman-Edelstein et al., 2014). The prevention and repair of GEnC injury have shown potential as a promising therapeutic target in DN (Ahotupa, 2017). As with other vascular endothelial cells, GEnCs can be directly affected by circulating substances, such as blood glucose, blood lipids, and inflammatory mediators. Researchers have observed cholesterol accumulation in the GEnCs of patients with DN and the cholesterol accumulation could lead to GEnCs injury accompanied by the production of a wide range of inflammatory cytokines *in vitro* (Yin et al., 2016).

Taken together, LncRNAs act as a bridge between high glucose (HG) and lipotoxicity induced by cholesterol accumulation in GEnCs, and an understanding of the role of glucolipid metabolism disorders on the expression of ABCA1 and cholesterol accumulation in GEnCs via a regulatory pathway involving LncRNAs may uncover the underlying mechanisms of the pathogenesis of DN.

## Materials and Methods

### Cell Culture

GEnCs (SienCell, United States) were cultured in endothelial cell medium (ECM) (SienCell, United States) supplemented with endothelial cell growth factors (SienCell, United States), 5% FBS, and 1% penicillin/streptomycin. The GEnCs were maintained in a humidified incubator containing 5% CO_2_ at 37°C and were divided into a Control Group [ECM with low glucose (LG), 5.6 mmol/L], HG Group (ECM with HG, 25 mmol/L), high cholesterol (HC) Group [ECM with HC, 400 μg/ml (carboxymethyl-β-cyclodextrin cholesterol (water-soluble cholesterol) (Sigma, United States)], and HG with HC Group (ECM with HG and HC). GW3965 (MedChemExpress, United States) was used to agitate the LXRs in the GEnCs.

### Oil Red O Staining

After treatment with cholesterol or glucose for 24 h, each group was rinsed in PBS, fixed in 4% paraformaldehyde for 30 min, stained in freshly diluted oil red O (Solarbio, Beijing, China) for 15 min, decolorized in 70% ethanol solution for 15 s, re-dyed in hematoxylin (Solarbio, Beijing, China) staining solution for 30 s, and rinsed in PBS twice. Then, the cells were observed and photographed under an inverted microscope. Image J pro plus software was used to calculate the area of oil red O staining.

### Quantification of Total Cholesterol

Total cholesterol levels in the GEnCs were measured using enzymatic colorimetric assay. In brief, the cells were collected from six-well plates and then crushed using ultrasonic waves. Subsequently, the total cholesterol levels of the cells were determined using a total cholesterol assay kit (Jiancheng Bioengineering, Nanjing, China) according to the manufacturer’s instructions. Then, the absorbance of the extraction was measured at 510 nm using a microplate reader.

### Immunofluorescence

Immunofluorescence staining was performed to detect the expression of ABCA1 in the GEnCs. In brief, the cells were fixed with 4% paraformaldehyde for 30 min and then blocked with 5% goat serum for 30 min. Subsequently, the cells were incubated with an anti-ABCA1 (1:200, Abcam) antibody at 4°C overnight, followed by incubation with FITC goat anti-rat IgG (H+L) secondary antibody (Sungene Biotech, Tianjin, China) in the dark for 1 h. The nuclei were stained with 4’,6-diamidino-2-phenylindole (Beyotime, Shanghai, China) for 5 min. The cells were observed under a fluorescence microscope.

### Measurement Using the Cell Counting Kit-8 (CCK-8)

CCK-8 assay (Solarbio, Beijing, China) was used to assess cell viability. The GEnCs were exposed to different concentrations of cholesterol (200, 400, and 600 μg/ml). After the ECM was replaced, 10 μl of CCK-8 reagent was added to a 96-well plate containing the cells, which was incubated in the dark at 37°C for 1–4 h. Absorbance was measured at 450 nm using a microplate reader (Tecan). All experiments were repeated three times.

### Cell Transfection

A LncOR13C9-pcDNA3.1 plasmid and control plasmid (GenePharma, Shanghai, China), siRNAs targeting LncOR13C9, and its negative control RNA (si-NC) (GenePharma, Shanghai, China) were generated. The siRNA sequences are shown in Table 1. The GEnCs were transfected using a Lipofectamine 2000 system (Thermo Fisher Scientific) according to the manufacturer’s instructions.

**TABLE 1:** The primer sequences for qRT-PCR.

ABCA1	5′-GCAGGCAATCATCAGGGTGC-3′	5′-TTCAGCCGTGCCTCCTTCTC-3′
LDLR	5′-AGGAGACGTGCTTGTCTGTC-3′	5′-CTGAGCCGTTGTCGCAGT-3′
LXR-α	5′-AGGGCTGCAAGGGATTCTTCC-3′	5′-TCTGACAGCACACACTCCTCCC-3
LXR-β	5′-CTCAGTCCAGGAGATCGTGG-3′	5′-CACTCTGTCTCGTGGTTGTAG-3′
RXR-α	5′-AAGATGCGGGACATGCAGAT-3′	5′-CGAGAGCCCCTTGGAGTCA-3′
RXR-β	5′-CCGATCCATTGATGTTCGAGAT-3′	5′-TCTGTCAGCACCCGATCAAAG-3′
FXR	5′-CCAACCTGGGTTTCTACCC-3′	5′-CACACAGCTCATCCCCTTT-3′
SREBP2	5′-GAGACCATGGAGACCCTCAC-3′	5′-GGAGCTACACAGCTGTTCTGA-3′
NONHSAT132788	5′-GGCAGAGACAGCCACAAGGAATTC-3′	5′-GCCAGTGAGCTGAGTGTCTGATG-3′
NONHSAT133801	5′-AGGTAGAGAAGCTGGACCAACTGG-3′	5′-AGCAATGCAAGGCATGTCAATGTC-3′
NONHSAT134718	5′-GGCTGCCACCACCATCATAATCC-3′	5′-TCTCCACTCACTGCAACCTCTCC-3′
NONHSAT135720	5′-GGCATCCTTGTCTTGTTGCAGTTC-3′	5′-CCTCAACCAACTAGGCTTGGAAGG-3
LINC00092	5′-CCTATGATTTGGCCTCTGGA-3′	5′-GAGAGCAGCGTTCAGGAAAC-3′
LncRNAOR13C9 si-RNA-1	5′-GGUAGAGAAGCUGGACCAATT-3′	5′-UUGGUCCAGCUUCUCUACCTT-3′
LncRNAOR13C9 si-RNA-2	5′-GGGCCCUGUUAGCUGACAUTT-3′	5′-AUGUCAGCUAACAGGGCCCTT-3′
LncRNAOR13C9 si-RNA-3	5′-GCCUCUAAACUCAGGGUUUTT-3′	5′-AAACCCUGAGUUUAGAGGCTT-3′
LncRNAOR13C9 si-RNA-4	5′-CCAUCUGCCUCUUUCAAUATT-3′	5′-UAUUGAAAGAGGCAGAUGGTT-3′
GAPDH	5′-ATGGGGAAGGTGAAGGTCG-3′	5′-GGGGTCATTGATGGCAACAATA-3′

### RNA Isolation and Quantitative Real-Time PCR Analyses

Total RNAs were extracted from cells using TRIzol reagent (Invitrogen, Waltham, United States), and cDNA was synthesized from RNA using a Reverse Transcription Kit (Takara, Beijing, China). qRT-PCR reactions were conducted using the SYBR Green Master (Takara, Beijing, China) along with a CFX96 real-time PCR detection system (Bio-Rad, United States). The primer sequences used are shown in Table 1. Fold change difference in gene expression was calculated using the 2^–Δ^ ^Δ^ ^Ct^ method.

### Western Blotting Analysis

The treated cells were washed with PBS and lysed in a RIPA lysis buffer (Beyotime, Shanghai, China) supplemented with a protease inhibitor cocktail (Boster, Wuhan, China) to extract total proteins. The membrane protein was extracted from the cells using a Membrane Protein Extraction Kit (Beyotime, Shanghai, China). Immunoblotting analysis was performed as previously described (Yin et al., 2016) following standard procedures and using the primary antibodies, ABCA1, LDLR (Abcam), LXR (ABclonal), and Anti-β-actin (ProteinTech). In brief, the protein was separated on an 8–10% SDS denatured polyacrylamide gel and then transferred onto a NC membrane. The membranes were blocked with 5% skim milk and were incubated with appropriate antibodies at 4°C overnight. Then, the membranes were washed and incubated with horseradish peroxidase–conjugated secondary antibodies (1:3,000; Sanjian, Tianjin, China). The protein of interest was visualized using an Immobilon Western Chemiluminescent HRP Substrate (Thermo Fisher Scientific).

### Fluorescence *in situ* Hybridization (FISH)

The prepared cell samples were fixed with 5% formaldehyde for 25 min. Then, the cells were incubated with a LncRNAOR13C9 probe (GenePharma, Shanghai, China) at 42°C overnight. After the sample was washed with SSC solution, the nuclei of the cells were stained with DAPI. The stained results were observed using a confocal laser scanning microscope.

### Microarray Analysis

Total RNAs were extracted from GEnCs using TRIzol reagent (Invitrogen, Waltham, United States). LncRNA microarray analysis was performed using Affymetrix Arrays (CapitalBio Corporation, Beijing, China). Microarray hybridization was performed based on standard protocols for Affymetrix assay. In brief, all RNA samples were amplified and transcribed, labeled, hybridized, and washed using GeneChip WT Terminal Labeling, Controls Kit, Ambion WT Expression Kit, and GeneChip Hybridization, Wash, and Stain Kit, respectively. The microarray analysis was performed using Affymetrix GeneChip Operating Software and scanned using the GeneChip Scanner 3000 7G.

### Luciferase Assay

The wild-type LncOR13C9 untranslated region (UTR) and a mutant LncOR13C9 UTR were cloned into the pmirGLO luciferase reporter plasmid. Luciferase vectors were transfected with the miR-23a-5p mimics or negative control and into HEK-293T cells using a Lipofectamine 2000 system. Forty-eight hours after transfection, the cells were collected and lysed. A Dual Luciferase Reporter Assay kit (Beyotime, Shanghai, China) was used to detect relative luciferase activity. Thereafter, firefly luciferase and renilla luciferase activities of the cell lysates were measured. Renilla luciferase activity was used as a standard for variations in transfection efficiency.

### Statistical Analysis

SPSS software was used for all statistical analyses, and *t*-test was used for data analysis and comparison between two groups. Data on three or more groups were analyzed using one-way ANOVA, and data were presented as $\bar{\text{x}}$ ± s. A *P*-value of <0.05 indicated statistical significance.

## Results

### HG Exacerbates Intracellular Cholesterol Accumulation Under Cholesterol Load

**FIGURE 1:**
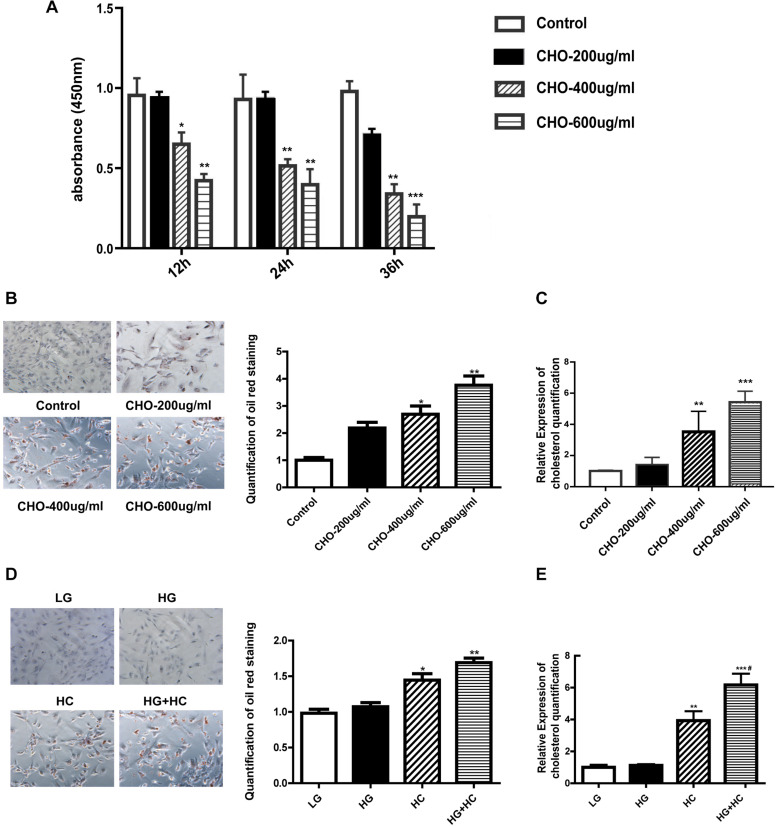
HG exacerbates intracellular cholesterol accumulation under cholesterol load. **(A)** CCK-8 assay was used to evaluate the influence of water-soluble cholesterol on GEnC viability (*P < 0.05 vs. Control; **P < 0.01 vs. Control). **(B)** Oil red O staining of GEnCs treated with different concentrations of cholesterol (*P < 0.05 vs. Control; **P < 0.01 vs. Control). **(C)** Cholesterol quantification experiment was conducted to determine the content of intracellular cholesterol (**P < 0.01 vs. Control; ***P < 0.001 vs. Control). **(D)** Oil red O staining of GEnCs treated with different concentrations of cholesterol and glucose (*P < 0.05 vs. LG; **P < 0.01 vs. LG). **(E)** Cholesterol quantification experiment was conducted to determine the content of intracellular cholesterol (**P < 0.01 vs. LG; ***P < 0.001 vs. LG; ^#^*P* < 0.05 vs. HC). CHO, water-soluble cholesterol.

Water-soluble cholesterol was used to establish a suitable HC model in the GEnCs. We determined cell viability under different cholesterol concentrations using CCK-8 assay, and the results showed that the viability of cells grown in HC decreased in a time-dependent and concentration-dependent manner, compared with that of cells grown under normal control condition. Thereafter, we established that 400 μg/ml cholesterol at 24 h was a suitable cholesterol intervention condition to explore lipotoxicity induced by cholesterol accumulation (Figure 1A). Oil red O staining was carried out to observe cholesterol accumulation in GEnCs and the results showed that intracellular cholesterol accumulation in cells grown in HC increased in a concentration-dependent manner (Figure 1B). The results of cholesterol quantification further supported these observations (Figure 1C). To evaluate the effect of HG on cholesterol accumulation in cells, oil red O staining was performed and the results showed that cholesterol accumulation was not remarkable after treatment with HG compared with the normal control, but increased under HC condition, compared with the normal control and HG groups. Intracellular cholesterol accumulation in cells treated with HG and HC was significantly higher than that with HG or HC alone (Figure 1D). These results were further confirmed through cholesterol quantification (Figure 1E). Overall, these results demonstrated that HG may aggravate intracellular cholesterol accumulation under cholesterol load.

### HG Interferes With Cell Responses to Excess Cholesterol by Changing the Expressions of ABCA1 and Low-Density Lipoprotein Receptor (LDLR)

**FIGURE 2:**
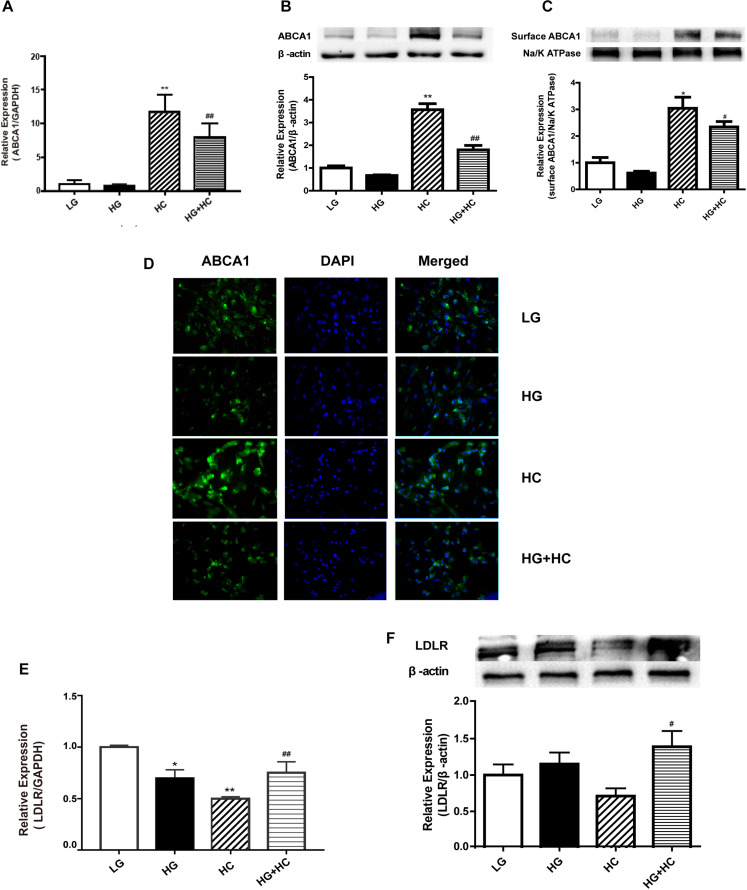
HG interferes with cell responses to excess cholesterol by affecting the expressions of ABCA1 and low-density lipoprotein receptor (LDLR). **(A)** RT-qPCR analysis to determine ABCA1 mRNA expression (**P < 0.01 vs. LG; ^##^*P* < 0.01 vs. HC). **(B,C)** WB analysis to determine total ABCA1 and surface protein expression (*P < 0.05 vs. LG; ^#^*P* < 0.05 vs. HC). **(D)** Immunofluorescence experiment was conducted to determine the expression of ABCA1. **(E,F)** RT-qPCR and WB analyses were conducted to determine LDLR expression (*P < 0.05 vs. LG; **P < 0.01 vs. LG; ^#^*P* < 0.05 vs. HC; ^##^*P* < 0.01 vs. HC).

To explore the mechanism by which HG increased GEnCs cholesterol accumulation, the expressions of LDLR and ABCA1 in GEnCs treated with different concentrations of glucose and cholesterol were detected using Western blotting (WB) and RT-qPCR analyses. Compared with the normal control, ABCA1 mRNA and protein levels were downregulated in the HG group and significantly upregulated in the HC group. However, the expression of ABCA1 in the HG and HC groups were downregulated, compared with that in the HC group (Figures 2A–C). Immunofluorescence analysis further supported these results (Figure 2D). Furthermore, the RT-qPCR and WB analyses results showed that the expression of LDLR increased in cells under the HG condition and decreased in cells under the HC condition. Compared with the HC group, the expression of LDLR in cells of the HG and HC groups were significantly elevated (Figures 2E,F).

### HG Affects ABCA1 Upregulation Through LXRs Under Cholesterol Load

**FIGURE 3:**
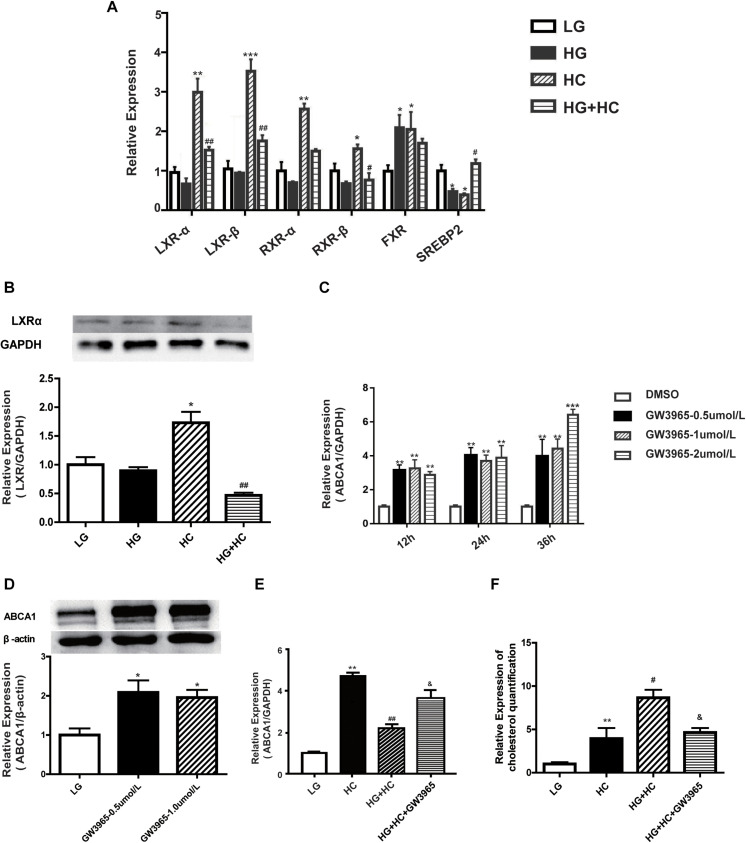
HG affects ABCA1 upregulation by LXRs under cholesterol load. **(A,B)** RT-qPCR analysis was conducted to determine the expression of transcription factors (*P < 0.05 vs. LG; **P < 0.01 vs. LG; ***P < 0.001 vs. LG; ^#^*P* < 0.05 vs. HC; ^##^*P* < 0.01 vs. HC). **(C,D)** RT-qPCR and WB analyses to determine ABCA1 expression at different concentrations of GW3965 (*P < 0.05 vs. LG; **P < 0.01 vs. LG; ***P < 0.001 vs. LG). **(E)** RT-qPCR analysis to determine the expression of ABCA1 in cells at HG and HC with GW3965 (**P < 0.01 vs. LG; ^##^*P* < 0.01 vs. HC; ^&^*P* < 0.05 vs. HG+HC). **(F)** Cholesterol quantification analysis to determine cholesterol accumulation in cells exposed to HG and HC conditions, along with GW3965 (**P < 0.01 vs. LG; ^#^*P* < 0.05 vs. HC; ^&^*P* < 0.05 vs. HG+HC).

To explore the mechanism by which HG affects the expression of ABCA1 under cholesterol load, four ABCA1-associated transcription factors (TFs) (LXRs, RXRs, FXR, and SREBP2) were identified from previous literature reports. LXRs bind to the retinoic X-receptor (RXRα) as obligate heterodimerization partners to direct repeats separated by four nucleotides (DR4-elements) in the promoter of ABCA1. SREBP2, which causes the downregulation of ABCA1, binds directly to the E-box of the ABCA1 promoter (Song et al., 1994; Edwards et al., 2002; Zeng et al., 2004; Lee and Tontonoz, 2015). The RT-qPCR results showed that expressions of LXRs and RXRs were downregulated in cells of the HG group, compared with the normal control. mRNA expression levels of these TFs significantly increased in cells of the HC group, compared with that of the normal control. However, compared with the HC group, the expressions of TFs in cells of the HG and HC groups were significantly downregulated. The expression of FXR mRNA increased in HC cells, compared with that of normal control cells. Compared with the HC group, no significant decrease was observed in FXR mRNA expression levels under HG and HC combined treatment conditions. Additionally, the expression of SREBP2 in cells grown in HC was significantly lower than that of the normal control. Compared with the HC group, cells of the HG and HC groups showed increased SREBP2 expression (Figures 3A,B). These findings suggested that LXRs and RXRs may be positive regulators of ABCA1 that cause the upregulation of ABCA1. Inversely, SREBP2 may cause the downregulation of ABCA1. Potentially involved LXRs were identified for further experimentation. The LXR agonist, GW3965, was used to determine whether LXRs could regulate the transcription of ABCA1 in GEnCs, and the results showed that the activation of LXRs by GW3965 significantly upregulated the expression of ABCA1 (Figures 3C,D). We further explored whether GW3965 could alter the expression of ABCA1 in cells under HG and HC combine treatment. The results showed that the expression of ABCA1 in cells under HG and HC treatment with GW3965 increased, compared to that without GW3965 (Figure 3E). In addition, the results of the cholesterol quantification proved that the downregulation of ABCA1 under HG and HC conditions enhanced cholesterol accumulation, but that activation of LXRs partially reversed cholesterol accumulation in GEnCs (Figure 3F).

### Analysis and Verification of LncRNA Microarray Results

**FIGURE 4:**
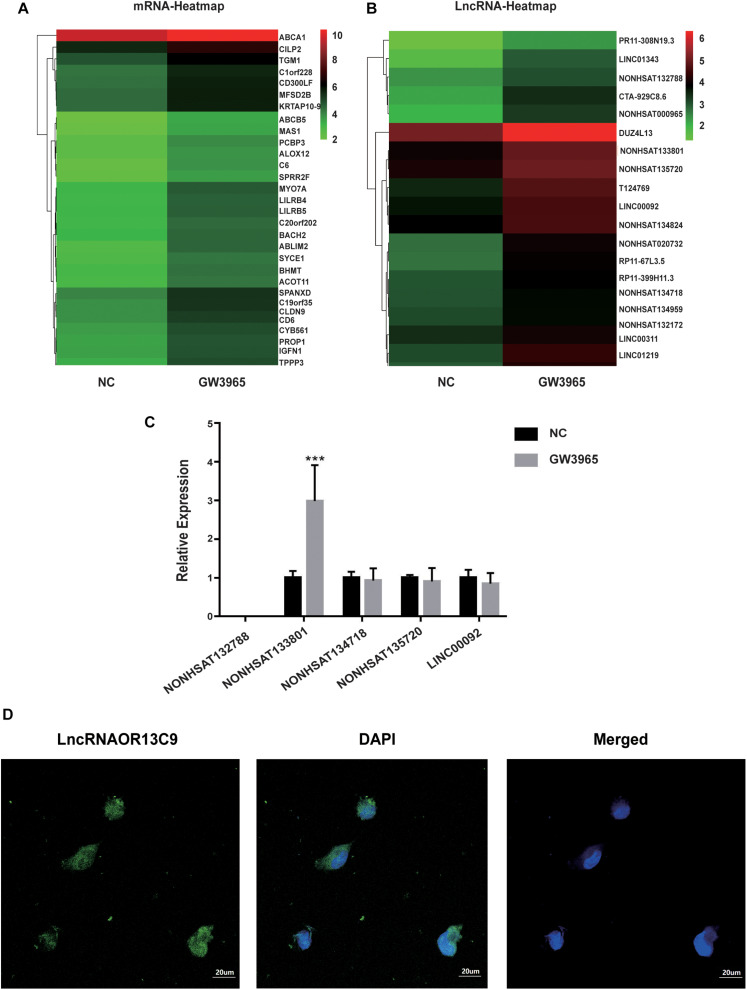
Analysis and verification of LncRNA microarray results. **(A,B)** The heatmap for the differential expression of mRNAs and LncRNAs under the influence of GW3965. **(C)** RT-qPCR analysis to determine the expression of five candidate LncRNAs (***P < 0.001 vs. NC). **(D)** The FISH for the location of LncRNAOR13C9 in GEnCs (magnification, 400×).

LncRNA microarray analysis results showed that compared with the control group, the GW3965 treatment group contained 2,116 mRNAs with a fold change value (FC value) of >1.5, and that among them, 1767 were upregulated and 349 were downregulated. Additionally, 2501 LncRNAs, including 1101 that were upregulated and 1400 that were downregulated, showed an FC value of >1.5 (Figures 4A,B). The increased expression of ABCA1 in cells grown with the LXR agonist indicated that LXRs could promote the transcription of ABCA1 in GEnCs. Previous studies have found that LncRNAs can function as molecular miRNA sponges involved in the regulation of target genes (Jia et al., 2016; Hennessy et al., 2019). Thus, we aimed to further explore whether any LncRNAs were involved in the process of ABCA1 regulation. Based on the FC values of the LncRNAs, five candidate LncRNAs (NONHSAT132788, NONHSAT133801, NONHSAT134718, NONHSAT135720, and LINC00092) were identified from among the upregulated LncRNAs to be used in further experiments. The RT-qPCR analysis results showed that NONHSAT133801 (LncRNAOR13C9) in the GW3965 treatment group was significantly upregulated (Figure 4C). However, even without the binding sites in the LncOR13C9 promoter, GW3965 could provoke LncOR13C9 expression. Using FISH, we found that LncRNAOR13C9 (LncOR13C9) was distributed in both the nucleus and the cytoplasm (Figure 4D). The localization of LncOR13C9 in the cytoplasm indicated the possibility that it may contain a translated open reading frame. However, we searched the long non-coding RNA database^1^ and found that LncOR13C9 did not perform a protein-coding function (Table 2). Therefore, the potential biological role of LncOR13C9 in GEnCs needs to be further explored.

**TABLE 2:** The protein coding potential of LncRNAOR13C9.

Metric	Raw result	Interpretation
PRIDE reprocessing 2.0	0	Non-coding
Lee translation initiation sites	0	Non-coding
PhyloCSF score	−138.2395	Non-coding
CPAT coding probability	0.93%	Non-coding
Bazzini small ORFs	0	Non-coding

### LncOR13C9 Is Involved in the Regulation of ABCA1

**FIGURE 5:**
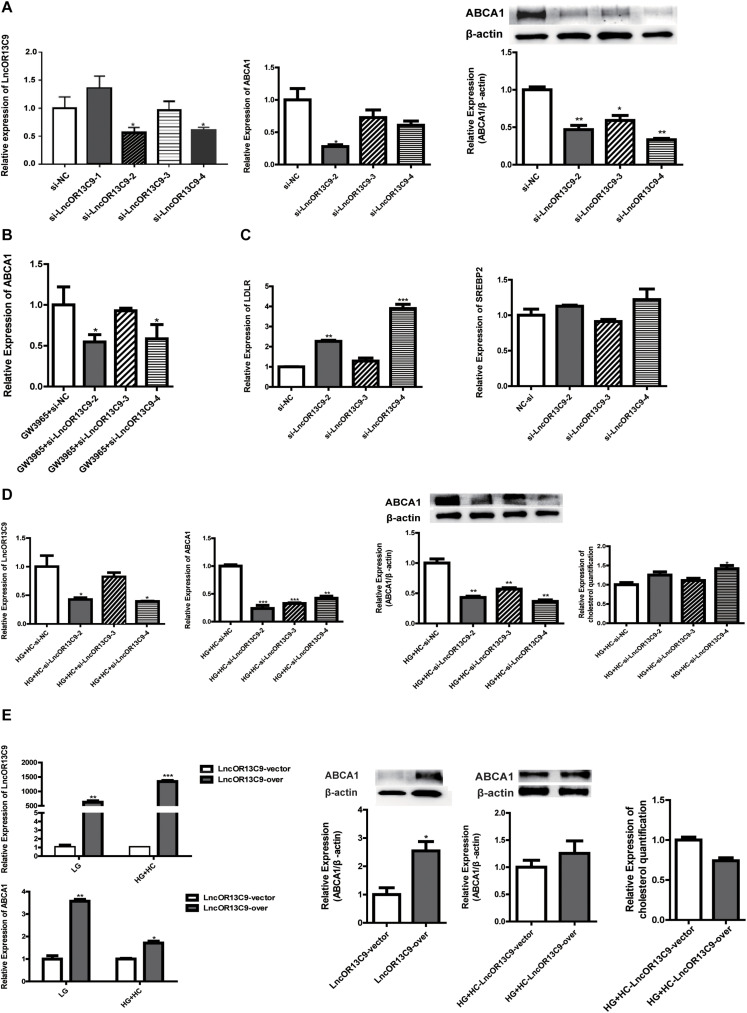
LncOR13C9 is involved in the regulation of ABCA1. **(A)** RT-qPCR and WB analyses to determine the knockdown efficiency of LncRNAOR13C9 and RT-qPCR and WB analyses to determine the expression of ABCA1 in cells transfected with siRNA-2,3,4 (*P < 0.05 vs. si-NC). **(B)** RT-qPCR analysis to determine ABCA1 expression in GEnCs treated with LncRNAOR13C9 siRNA and GW3965 (***P < 0.001 vs. LG; ^##^*P* < 0.05 vs. GW3965+si-NC). **(C)** RT-qPCR analysis to determine the LDLR and SREBP2 expressions in GEnCs treated with LncRNAOR13C9 siRNA (**P < 0.01 vs. si-NC; ***P < 0.001 vs. si-NC). **(D)** RT-qPCR and WB analyses to determine the expression of ABCA1 and cholesterol quantification analysis to determine cholesterol accumulation in cells grown under HG and HC conditions, along with LncRNAOR13C9 knockdown (*P < 0.05 vs. HG+HC si-NC; **P < 0.01 vs. HG+HC si-NC; ***P < 0.001 vs. HG+HC si-NC). **(E)** RT-qPCR analysis to determine the overexpression efficiency of LncRNAOR13C9 (*P < 0.05 vs. LncRNAOR13C9 vector; **P < 0.01 vs. LncRNAOR13C9 vector; ***P < 0.001 vs. LncRNAOR13C9 vector); RT-qPCR and WB analyses to determine the expression of ABCA1 in cells transfected with the LncOR13C9-pcDNA3.1 plasmid (*P < 0.05 vs. the LncRNAOR13C9 vector); and cholesterol quantification analysis to determine cholesterol accumulation in cells grown under HG and HC conditions, along with LncRNAOR13C9 overexpression.

We explored the role of LncOR13C9 in the LXR pathway that regulates cellular responses to excess cholesterol through ABCA1. To test the effect of LncOR13C9 on GEnCs, siRNAs targeting LncOR13C9, as well as LncRNAOR13C9 plasmids and empty vectors, were constructed and transfected into the cells. RT-qPCR analysis was conducted and the results showed that the expression of LncOR13C9 in cells transfected with siRNA-2,3,4 was lower than that with NC-si (Figure 5A). The knockdown of LncOR13C9 using siRNAs or combined with the LXRs agonist (GW3965) showed that the activation of LXRs could significantly increase the expression of ABCA1, but that the upregulation of ABCA1 significantly decreased the effect of GW3965 when LncOR13C9 was knocked down (Figure 5B). Furthermore, we found that the downregulation of LncOR13C9 could increase LDLR expression but did not alter SREBP2 expression (Figure 5C). We hypothesized that LncOR13C9 may be involved in the regulation of LDLR. We also showed that knockdown of LncOR13C9 in cells exposed to HG and HC conditions could decrease ABCA1 expression and aggravate cholesterol accumulation (Figure 5D). However, opposing effects were observed in cells in which LncOR13C9 was overexpressed (Figure 5E).

### The LncOR13C9 Regulates ABCA1 Expression by Regulating miR-23a-5p

**FIGURE 6:**
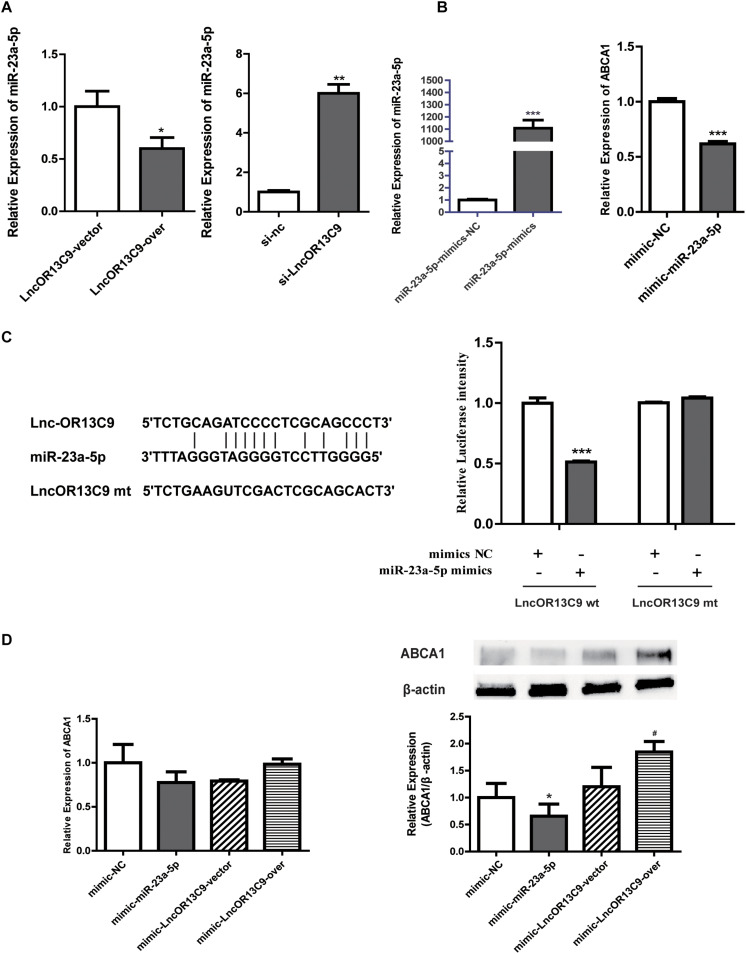
LncOR13C9 regulates ABCA1 expression through miR-23a-5p. **(A)** RT-qPCR analysis to determine miR-23a-5p expression with the overexpression and knockdown of LncOR13C9. **(B)** RT-qPCR analysis to determine the overexpression efficiency of miR-23a-5p and the expression of ABCA1 in cells transfected with the miR-23a-5p mimic. **(C)** Luciferase reporter assay on GEnCs co-transfected with LncOR13C9-3’UTR-WT or LncOR13C9-3’UTR-MUT and miR-23a-5p mimics or NC (***P < 0.001 vs. LncOR13C9-WT+NC group). **(D)** Rescue experiments to determine the regulatory effect of LncOR3C9/miR-23a-5p/ABCA1 in GEnCs (*P < 0.05 vs. mimic NC; ^#^*P* < 0.05 vs. miR-23a-5p mimic with LncOR13C9 vector).

The cytoplasmic localization of LncRNAOR13C9 indicated the possibility that it may interact with cytosolic miRNAs known to post-transcriptionally regulate ABCA1 expression. Recently, miRNAs, such as miR-33, miR-23a-5p, miR-27b, miR-33a, miR-33b, and miR-128, have emerged as potent regulators of cholesterol homeostasis that act through their ability to repress the expression of ABCA1. The bioinformatics analysis (RegRNA 2.0) showed that LncOR3C9 was a potential ceRNA of miR-23a-5p. To explore this potential mechanism, plasmids expressing LncOR3C9 or empty plasmids were transfected into GEnCs. The qPCR results showed that the overexpression of LncOR3C9 downregulated miR-23a-5p expression, compared with empty vector transfected cells. Conversely, knockdown of LncOR3C9 using siRNA increased miR-23a-5p expression (Figure 6A). We further verified that transfection with miR-23a-5p mimics downregulated ABCA1 expression (Figure 6B), which was also reported by Yang et al., who demonstrated that miR-23a-5p repressed 3’ UTR activity of ABCA1 (Yang et al., 2018). To investigate whether LncOR3C9 was a functional target of miR-23a-5p, dual-luciferase gene reporter assays were performed. We found that the luciferase activity in the LncOR3C9-3’ UTR-WT+miR-23a-5p mimics group was significantly lower, compared with the LncOR3C9-3’-WT+miR-23a-5p-NC group. No significant difference was observed in luciferase activity between the LncOR3C9-3’UTR-WT+miR-23a-5p mimics group and LncOR3C9-3’ UTR-WT+miR-23a-5p mimics group (Figure 6C). These results suggested that LncOR3C9 was a target of miR-23a-5p. To elucidate the regulatory effect of LncOR3C9/miR-23a-5p on ABCA1 expression in GEnCs, rescue assays were designed and performed. The results showed that LncOR3C9 overexpression abrogated the miR-23a-5p-mediated repressive effects of ABCA1 (Figure 6D).

## Discussion

This study investigated the role of HG on cholesterol accumulation in GEnCs. We found that HG downregulated LXRs expression in GEnCs under cholesterol load and that the decrease in LXRs expression suppressed ABCA1 expression and increased cholesterol accumulation. Furthermore, the downregulation of LXRs decreased LncOR13C9 expression, and knockdown of LncOR13C9 downregulated ABCA1 expression. Bioinformatics analysis and luciferase reporter assay found that LncOR13C9 mediated these effects via binding to miR-23a-5p.

The stability of cholesterol metabolism plays a crucial role in maintaining the functions of organisms. However, excessive cholesterol accumulation can impair cellular functions by inducing the release of inflammatory factors, oxidative stress, and apoptosis. Previous studies have found that cholesterol can cause the death of foam cells derived from macrophages, indicating that cholesterol can cause apoptosis by activating the mitochondrial apoptosis pathway and the endoplasmic reticulum apoptosis pathway (Bao et al., 2007). In our study, CCK-8 assay also showed that cholesterol could damage GEnCs at certain levels. The ability of cells to effectively respond to a changing environment provides protection from cholesterol overload. When cholesterol load exceeds the threshold at which cells are able to respond, excessive cholesterol cannot be metabolized and flow out from cells, but accumulates in cells, thereby causing the dysfunction of cells. Furthermore, we found that the ability to respond to cholesterol load was somewhat diluted by the addition of HG, resulting in an increase of cholesterol accumulation in GEnCs. To our knowledge, ABCA1 is a membrane protein that is associated with regulatory genes involved in response to excess cholesterol (Zhang et al., 2011; Li C. H. et al., 2017). Yin et al. (2016) found that the expression level of ABCA1 in apoE^–/–^ mice with diabetes was lower than that in apoE^–/–^ mice without diabetes and that the downregulation of ABCA1 in the kidneys of diabetic mice could lead to cholesterol accumulation, excessive production of cytoinflammatory cytokines and renal dysfunction, suggesting that ABCA1 dysregulation was closely associated with DN. In this study, we found that HG could decrease mRNA and protein levels of ABCA1 in GEnCs under HC, but this observation is controversial since some studies have reported conflicting results. Tsujita et al. (2017) concluded that ABCA1 protein expression was upregulated in the liver of diabetic animals without an apparent increase of its mRNA. Most others studies have found the mRNA and protein levels of ABCA1 decreased in the liver of diabetic mice, as well as in HepG2 or RAW264 cells exposed to HG (Tu and Albers, 2001; Uehara et al., 2002), a conclusion that is consistent with our results. However, no rational interpretation has been proposed for these discrepancies. Furthermore, we examined the effects of HG and HC on LDLR, a protein that mediates cholesterol flow into cells. The results showed that LDLR expression increased in cells treated with HG and HC, compared with that treated with HC alone. This was probably due to the downregulation of LDLR to prevent excessive cholesterol from entering the cells in response to cholesterol overload. However, HG interfered with the ability of the cells to respond to a changing environment, allowing more cholesterol to flow into cells and aggravate intracellular cholesterol accumulation.

At transcriptional level, LXRs are important for the regulation of genes that control the response to excess cholesterol (Lee and Tontonoz, 2015). Our results showed that the activation of LXRs could upregulate the expression of ABCA1 and partially reverse the decrease in ABCA1 expression caused by HG in cells treated with HC. Moreover, HG can aggravate intracellular cholesterol accumulation in cells grown in HC. This indicated that the regulatory pathway of LXRs/ABCA1 is involved in the process through which HG exacerbates cholesterol accumulation in GEnCs under conditions of excess HC.

Recent studies have shown that LncRNAs are involved in cholesterol metabolism (Sallam et al., 2016, 2018; Yan et al., 2016). However, the regulatory role of LncRNAs in the occurrence and development of DN and whether LncRNAs participate in cholesterol accumulation in GEnCs due to HG and HC are not clear. Five LncRNAs were selected from the gene microarray and identified through RT-qPCR analysis. The results showed that GEnCs cultured with the LXR agonist showed increased LncOR13C9 expression, compared with the vehicle treatment. Although we did not identify the direct binding sites of LXRs on the LncOR13C9 promoter, the LXR agonist, GW3965, could stimulate LncOR13C9 expression, indicating that LncOR13C9 plays a role in the transcription of LXRs. Moreover, our results showed that HC could upregulate LncOR13C9 expression, which was downregulated by HG. Knockdown of LncOR13C9 decreased ABCA1 expression and aggravated cholesterol accumulation in GEnCs treated with HG and HC. Reverse effects were observed in GEnCs with LncOR13C9 overexpression.

LncRNAs modulate biological processes, including chromatin remodeling, transcription, and post-transcription, via regulating gene expression (Edwards and Ferguson-Smith, 2007; Wan and Bartolomei, 2008; Lee, 2011; Shi et al., 2013). LncRNAs may act as molecular miRNA sponges that negatively modulate the expression of miRNAs to regulate post-transcription (Jia et al., 2016). Therefore, we hypothesized that LncOR13C9 may act as a miRNA sponge that binds to miRNAs in GEnCs to regulate ABCA1. In this study, through a bioinformatics analysis, we found that miR-23a-5p may act as a bridge between LncOR13C9 and ABCA1. Dual luciferase reporter assay demonstrated that miR-23a-5p could bind to LncOR13C9 in a sequence-specific manner. Recent studies have shown that miR-23a-5p repressed the activity of the 3’UTR of ABCA1 (Yang et al., 2018). Moreover, we illustrated that the overexpression of LncOR13C9 downregulated miR-23a-5p and upregulated the expression of ABCA1. We also found that LncOR13C9 overexpression could partially increase ABCA1 expression, even if miR-23a-5p was overexpressed, suggesting that LncOR13C9 regulated ABCA1 expression through miR-23a-5p. These results suggested that LncOR13C9 positively regulated the post-transcriptional expression of ABCA1 by sponging miR-23a-5p in GEnCs. However, HG suppressed the expression of LncOR13C9 by decreasing LXRs expression to promote cholesterol accumulation through the upregulation of miR-23a-5p and subsequent inhibition of ABCA1 expression. Therefore, we speculated that high blood glucose caused by DM could damage GEnCs by disrupting cholesterol metabolism. This indicated that GEnCs damage caused by cholesterol accumulation aggravated by HG under cholesterol load could be associated with the development and progression of DN.

There are certain limitations of this study. First, we did not explore the specific effect of cholesterol accumulation on GEnCs. Second, we were not able to find suitable literature that could help us explore other pathways, such as the SREBP2 and LDLR pathways, to obtain a complete understanding of the combined effects of HG and HC. Therefore, further studies are needed to address these issues and to confirm our findings.

## Conclusion

In conclusion, we determined the expression of LncOR13C9 in GEnCs and described its regulatory function in glucose and cholesterol metabolism. We also inferred that the impact of HG on cholesterol accumulation in GEnCs under cholesterol load may be mediated via LXRs/LncOR13C9/miR-23a-5p. The determination of LncOR13C9 expression not only indicated the importance of identifying new regulatory mechanisms to enhance our understanding of glucose and cholesterol metabolic disorders involved in GEnCs damage, but may also enable the identification of new therapeutic strategies and targets for the prevention and treatment of DN.

## Data Availability Statement

The original contributions presented in the study are publicly available. This data can be found here: https://figshare.com/articles/dataset/GW3965_vs_NC_xlsx/12933188/1.

## Author Contributions

YW, SX, and PY originated and designed the study. YW, SX, and YL conducted the study, performed the analysis, and wrote the manuscript. YW revised the manuscript. SZ, RZ, and YL supervised the study and critically reviewed the manuscript for important intellectual content. All authors contributed to the article and approved the submitted version.

## Conflict of Interest

The authors declare that the research was conducted in the absence of any commercial or financial relationships that could be construed as a potential conflict of interest.

## Footnotes

1 http://www.lncipedia.org

## References

[B1] Ahotupa M. (2017). Oxidized lipoprotein lipids and atherosclerosis. *Free Radic. Res.* 51 439–447. 10.1080/10715762.2017.1319944 28412863

[B2] Attie A. D. Kastelein J. P. P. Hayden M. R. (2001). Pivotal role of ABCA1 in reverse cholesterol transport influencing HDL levels and susceptibility to atherosclerosis. *J. Lipid Res.* 42 1717–1726. 11714841

[B3] Bao S. Li Y. Lei X. Wohltmann M. Jin W. Bohrer A. (2007). Attenuated free cholesterol loading-induced apoptosis but preserved phospholipid composition of peritoneal macrophages from mice that do not express group VIA phospholipase A2. *J. Biol. Chem.* 282 27100–27114. 10.1074/jbc.m701316200 17627946 PMC2044506

[B4] Boivin V. Deschamps-Francoeur G. Couture S. Nottingham R. M. Bouchard-Bourelle P. Lambowitz A. M. (2018). Simultaneous sequencing of coding and noncoding RNA reveals a human transcriptome dominated by a small number of highly expressed noncoding genes. *RNA* 24 950–965. 10.1261/rna.064493.117 29703781 PMC6004057

[B5] Broekhuizen L. N. Lemkes B. A. Mooij H. L. Meuwese M. C. Verberne H. Holleman F. (2010). Effect of sulodexide on endothelial glycocalyx and vascular permeability in patients with type 2 diabetes mellitus. *Diabetologia* 53 2646–2655. 10.1007/s00125-010-1910-x 20865240 PMC2974920

[B6] Byron A. Randles M. J. Humphries J. D. Mironov A. Hamidi H. Harris S. (2014). Glomerular cell cross-talk influences composition and assembly of extracellular matrix. *J. Am. Soc. Nephrol.* 25 953–966. 10.1681/asn.2013070795 24436469 PMC4005312

[B7] Choby B. (2017). Diabetes update: prevention and management of diabetes complications. *FP Essent.* 456 36–40. 28530383

[B8] Das S. Reddy M. A. Senapati P. Stapleton K. Lanting L. Wang M. (2018). Diabetes mellitus-induced long noncoding RNA Dnm3os regulates macrophage functions and inflammation via nuclear mechanisms. *Arterioscler. Thromb. Vasc. Biol.* 38 1806–1820. 10.1161/atvbaha.117.310663 29930005 PMC6202204

[B9] Declèves A. E. Zolkipli Z. Satriano J. Wang L. Nakayama T. Rogac. (2014). Regulation of lipid accumulation by AMP-activated kinase [corrected] in high fat diet-induced kidney injury. *Kidney Int.* 85 611–623. 10.1038/ki.2013.462 24304883 PMC4244908

[B10] Edwards C. A. Ferguson-Smith A. C. (2007). Mechanisms regulating imprinted genes in clusters. *Curr. Opin. Cell Biol.* 19 281–289. 10.1016/j.ceb.2007.04.013 17467259

[B11] Edwards P. A. Kast H. R. Anisfeld A. M. (2002). BAREing it all: the adoption of LXR and FXR and their roles in lipid homeostasis. *J. Lipid Res.* 43 2–12. 10.1194/jlr.s087452 11792716

[B12] Gaemers I. C. Stallen J. M. Kunne C. Wallner C. van Werven J. Nederveen A. (2011). Lipotoxicity and steatohepatitis in an overfed mouse model for non-alcoholic fatty liver disease. *Biochim. Biophys. Acta* 1812 447–458. 10.1016/j.bbadis.2011.01.003 21216282

[B13] Hennessy E. J. Solingen C. V. Scacalossi K. R. Ouimet M. Afonso M. S. Prins J. (2019). The long noncoding RNA CHROME regulates cholesterol homeostasis in primates. *Nat. Metab.* 1 98–110. 10.1038/s42255-018-0004-9 PMC6691505 31410392

[B14] Herman-Edelstein M. Scherzer P. Tobar A. Levi M. Gafter U. (2014). Altered renal lipid metabolism and renal lipid accumulation in human diabetic nephropathy. *J. Lipid Res.* 55 561–572. 10.1194/jlr.p040501 24371263 PMC3934740

[B15] Jia P. Cai H. Liu X. Chen J. Ma J. Wang P. (2016). Long non-coding RNA H19 regulates glioma angiogenesis and the biological behavior of glioma-associated endothelial cells by inhibiting microRNA-29a. *Cancer Lett.* 381 359–369. 10.1016/j.canlet.2016.08.009 27543358

[B16] Kohlwein S. D. Petschnigg J. S. (2007). Lipid-induced cell dysfunction and cell death: lessons from yeast. *Curr. Hypertens. Rep.* 9 455–461. 10.1007/s11906-007-0084-5 18367008

[B17] Lawrie C. H. Armesto M. Fernandez-Mercado M. Arestín M. Manterola L. Goicoechea I. (2018). Noncoding RNA expression and targeted next-generation sequencing distinguish tubulocystic renal cell carcinoma (TC-RCC) from other renal neoplasms. *J. Mol. Diagn.* 20 34–45. 10.1016/j.jmoldx.2017.09.002 29056573

[B18] Lee J. T. (2011). Gracefully ageing at 50, X-chromosome inactivationbecomes a paradigm for RNA and chromatin control. *Nat. Rev. Mol. Cell Biol.* 12 815–826. 10.1038/nrm3231 22108600

[B19] Lee S. D. Tontonoz P. (2015). Liver X receptors at the intersection of lipid metabolism and atherogenesis. *Atherosclerosis* 242 29–36. 10.1016/j.atherosclerosis.2015.06.042 26164157 PMC4546914

[B20] Li C. H. Gong D. Chen L. Y. Wang L. Zhang W. (2017). Puerarin promotes ABCA1-mediated cholesterol efflux and decreases cellular lipid accumulation in THP-1 macrophages. *Eur. J. Pharmacol.* 811 74–86. 10.1016/j.ejphar.2017.05.055 28576406

[B21] Li D. Cheng M. Niu Y. Chi X. Liu X. Fan J. (2017). Identification of a novel human long non-coding RNA that regulates hepatic lipid metabolism by inhibiting SREBP-1c. *Int. J. Biol. Sci.* 13 349–357. 10.7150/ijbs.16635 28367099 PMC5370442

[B22] Liu P. Peng L. Zhang H. Tang P. M. Zhao T. Yan M. (2018). Tangshen formula attenuates diabetic nephropathy by promoting ABCA1-mediated renal cholesterol efflux in db/db mice. *Front. Physiol.* 9:343. 10.3389/fphys.2018.00343 29681863 PMC5897509

[B23] Matsuda J. Namba T. Takabatake Y. Kimura T. Takahashi A. Yamamoto T. (2018). Antioxidant role of autophagy in maintaining the integrity of glomerular capillaries. *Autophagy* 14 53–65. 10.1080/15548627.2017.1391428 29130363 PMC5846506

[B24] Murea M. Freedman B. I. Parks J. S. Antinozzi P. A. Elbein S. C. Ma L. (2010). Lipotoxicity in diabetic nephropathy: the potential role of fatty acid oxidation. *Clin. J. Am. Soc. Nephrol.* 5 2373–2379. 10.2215/cjn.08160910 21051750

[B25] Oram J. F. Lawn R. M. (2001). ABCA1. The gatekeeper for eliminating excess tissue cholesterol. *J. Lipid Res.* 42 1173–1179. 11483617

[B26] Palazzo A. F. Lee E. S. (2015). Non-coding RNA: what is functional and what is junk? *Front. Genet.* 6:2. 10.3389/fgene.2015.00002 25674102 PMC4306305

[B27] Plötz T. Hartmann M. Lenzen S. Elsner M. (2016). The role of lipid droplet formation in the protection of unsaturated fatty acids against palmitic acid induced lipotoxicity to rat insulin-producing cells. *Nutr. Metab.* 13:16. 10.1186/s12986-016-0076-z PMC4766664 26918025

[B28] Sallam T. Jones M. Thomas Brandon J. Wu X. Gilliland T. Qian K. (2018). Transcriptional regulation of macrophage cholesterol efflux and atherogenesis by a long noncoding RNA. *Nat. Med.* 24 304–312. 10.1038/nm.4479 29431742 PMC5839972

[B29] Sallam T. Jones M. C. Thomas G. Zhang L. Wu X. Eskin A. (2016). Feedback modulation of cholesterol metabolism by the lipid-responsive non-coding RNA LeXis. *Nature* 534 124–128. 10.1038/nature17674 27251289 PMC4896091

[B30] Schmitz S. U. Grote P. Herrmann B. G. (2016). Mechanisms of long noncoding RNA function in development and disease. *Cell Mol. Life Sci.* 73 2491–2509. 10.1007/s00018-016-2174-5 27007508 PMC4894931

[B31] Shapiro H. Theilla M. Attal-Singer J. Singer P. (2011). Effects of polyunsaturated fatty acid consumption in diabetic nephropathy. *Nat. Rev. Nephrol.* 7 110–121. 10.1038/nrneph.2010.156 21135888

[B32] Shi X. Sun M. Liu H. Yao Y. Song Y. (2013). Long non-coding RNAs:a new frontier in the study of human diseases. *Cancer Lett.* 339 159–166. 10.1016/j.canlet.2013.06.013 23791884

[B33] Siddiqi H. K. Kiss D. Rader D. (2015). HDL-cholesterol and cardiovascular disease: rethinking our approach. *Curr. Opin. Cardiol.* 30 536–542. 10.1097/hco.0000000000000211 26192490

[B34] Song C. Kokontis J. M. Hiipakka R. A. Liao S. (1994). Ubiquitous receptor: a receptor that modulates gene activation by retinoic acid and thyroid hormone receptors. *Proc. Natl. Acad. Sci. U.S.A.* 91 10809–10813. 10.1073/pnas.91.23.10809 7971966 PMC45115

[B35] Talkish J. May G. Lin Y. Woolford J. L. (2014). McManus CJ. Mod-seq: high-throughput sequencing for chemical probing of RNA structure. *RNA* 20 713–720. 10.1261/rna.042218.113 24664469 PMC3988572

[B36] Tsujita M. Hossain M. A. Lu R. Tsuboi T. Okumura-Noji K. Yokoyama S. (2017). Exposure to high glucose concentration decreases cell surface ABCA1 and HDL biogenesis in hepatocytes. *J. Atheroscler. Thromb.* 24 1132–1149. 10.5551/jat.39156 28428480 PMC5684479

[B37] Tu A. Y. Albers J. J. (2001). Glucose regulates the transcription of human genes relevant to HDL metabolism: responsive elements for peroxisome proliferator-activated receptor are involved in the regulation of phospholipid transfer protein. *Diabetes* 50 1851–1856. 10.2337/diabetes.50.8.1851 11473048

[B38] Uehara Y. Engel T. Li Z. Goepfert C. Rust S. Zhou X. (2002). Polyunsaturated fatty acids and acetoacetate downregulate the expression of the ATP-binding cassette transporter A1. *Diabetes Metab. Res. Rev.* 51 2922–2928. 10.2337/diabetes.51.10.2922 12351428

[B39] Wan L. B. Bartolomei M. S. (2008). Regulation of imprinting in clusters: noncoding RNAs versus insulators. *Adv. Genet.* 61 207–223. 10.1016/s0065-2660(07)00007-7 18282507

[B40] Wellington C. L. Walker E. K. Suarez A. Kwok A. Bissada N. Singaraja R. (2002). ABCA1 mRNA and protein distribution patterns predict multiple different roles and levels of regulation. *Lab. Investig.* 82 273–283. 10.1038/labinvest.3780421 11896206

[B41] Xiao S. Wang Y. Zhou S. Zhang R. Liu H. Lin Y. (2020). High glucose aggravates lipid deposition in glomerular endothelial cells by LXRs/LncRNAOR13C9/ABCA1 pathway. *Res. Square* 10.21203/rs.3.rs-17912/v1

[B42] Yan C. Chen J. Chen N. (2016). Long noncoding RNA MALAT1 promotes hepatic steatosis and insulin resistance by increasing nuclear SREBP-1c protein stability. *Sci. Rep.* 6:22640. 10.1038/srep22640 PMC4776244 26935028

[B43] Yang S. Ye Z. M. Chen S. Luo X. Y. X. Chen S. L. Mao L. (2018). MicroRNA-23a-5p promotes atherosclerotic plaque progression and vulnerability by repressing ATP-binding cassette transporter A1/G1 in macrophages. *J. Mol. Cell Cardiol.* 123 139–149. 10.1016/j.yjmcc.2018.09.004 30227118

[B44] Yin Q. H. Zhang R. Wang Y. T. Liu J. P. Zhang J. (2016). Exendin-4 ameliorates lipotoxicity-induced glomerular endothelial cell injury by improving ABC transporter A1-mediated cholesterol efflux in diabetic apoE knockout mice. *J. Biol. Chem.* 291 26487–26501. 10.1074/jbc.m116.730564 27784780 PMC5159509

[B45] Zeng L. Liao H. Liu Y. Lee T. S. Zhu M. Wang X. (2004). Sterol-responsive element-binding protein (SREBP) 2 down-regulates ATP-binding cassette transporter A1 in vascular endothelial cells: a novel role of SREBP in regulating cholesterol metabolism. *J. Biol. Chem.* 279 48801–48807. 10.1074/jbc.m407817200 15358760

[B46] Zhang G. Li Q. Wang L. Chen Y. Wang L. Zhang W. (2011). Interleukin-1beta enhances the intracellular accumulation of cholesterol by up-regulating the expression of low-density lipoprotein receptor and 3-hydroxy-3-methylglutaryl coenzyme A reductase in podocytes. *Mol. Cell. Biochem.* 346 197–204. 10.1007/s11010-010-0605-4 20936497

[B47] Zhao Y. F. Wang Z. Q. Yang J. Wang L. M. Zhao Z. P. Zeng X. Y. (2018). [Prevalence, awareness, status of treatment and control on type 2 diabetes mellitus among Chinese premenopausal women aged 18-49 in 2013]. *Zhonghua liu xing bing xue za zhi.* 39 213–217. 29495208 10.3760/cma.j.issn.0254-6450.2018.02.015

[B48] Zhu X. Lee J. Y. Timmins J. M. Brown J. M. Boudyguina E. Mulya A. (2008). Increased cellular free cholesterol in macrophage-specific Abca1 knock-out mice enhances pro-inflammatory response of macrophages. *J. Biol. Chem.* 283 22930–22941. 10.1074/jbc.m801408200 18552351 PMC2516976

